# Total intravenous anaesthesia in rural sub-Saharan Africa: report of 25 cases

**DOI:** 10.4314/ahs.v23i4.62

**Published:** 2023-12

**Authors:** Marrero-Garcia Ramses, Clusella Aleix, Prendes Diego, Soliva-Domínguez Ramón, Vives Teresa

**Affiliations:** 1 Hospital Clinic de Barcelona, Anesthesia and Reanimation; 2 Sant Joan de Deu Hospital, Anestesiología y Reanimación; 3 Joan XXIII University Hospital in Tarragona, Anestesiología y Reanimación; 4 Hospital Universitari Sagrat Cor, Servicio de Cirugía General y Digestiva (UNICIR SLP); 5 Hospital Universitario Quironsalud Madrid, Cirugía Torácica

**Keywords:** Intravenous drug administration, Anesthesia, Thyroidectomy

## Abstract

Total intravenous anesthesia (TIVA) is a potential solution for safe and effective anesthesia administration in rural regions of sub-Saharan Africa, where access to inhalational anesthesia may be limited. However, challenges such as equipment and medication availability, as well as a shortage of trained anesthesiologists, can limit the use of TIVA. In this study, the safety and efficacy of TIVA were evaluated in a case series of 25 female patients undergoing thyroid surgery in a rural area of sub-Saharan Africa. The TIVA protocol involved the use of propofol, fentanyl, rocuronium, and sugammadex. Results showed that TIVA was a safe and effective method of anesthesia administration in this setting, with no major adverse events reported. The use of TIVA may offer advantages such as faster recovery times, reduced incidence of postoperative nausea and vomiting, and reduced risk of environmental pollution compared to inhalational anesthesia. However, the cost and monitoring requirements of TIVA may pose challenges in resource-limited settings. Further research is needed to determine the optimal use of TIVA in this context.

## Introduction

Surgery is an important part of health care and is often the only option for treating certain conditions. Anesthesia is an important part of surgical care and safe anesthesia management is essential for a good surgical outcome. Access to anesthesia services is often limited in rural areas of sub-Saharan Africa and in many cases inhalational anesthesia is the only available option 1. Halothane has been widely used as an inhalation anesthetic for many years but is rarely used today due to potential risks and side effects. Halothane has been associated with hepatotoxicity that can cause severe and fatal liver necrosis. 2-4

According to reports the incidence of liver damage due to halothane is as high as one in ten thousand. Additionally, halothane has been linked to cardiac arrhythmias that can lead to serious cardiovascular events such as heart attacks 4. This potential risk combined with the availability of safer anesthetics has led to a decline in the use of halothane in clinical practice.

However, inhalational anesthesia has several limitations, including the need for specialized equipment and trained personnel, as well as potential adverse effects on patients and healthcare workers. Total intravenous anesthesia (TIVA) is an alternative method that can be administered with minimal equipment and training, making it a potentially valuable tool for anesthesia administration in rural areas.

Total intravenous anesthesia (TIVA) is a commonly used method of anesthesia in many parts of the world, including Africa. However, there are several challenges to using TIVA in this context. One of the main challenges is the lack of availability of the necessary equipment and medications. TIVA requires specialized infusion pumps and continuous monitoring of drug effects, such as proper haemodynamic monitoring and, probably, an electroencephalography measurement monitor, which may not be available in many rural areas of Africa 1. Additionally, there may be a shortage of the drugs used in TIVA, such as propofol, which can limit the use of this technique.

Another problem with the use of TIVA in Africa is the lack of skilled anesthesiologists 5. Anesthesiology is a relatively new field in many African countries and there may be a shortage of physicians trained to administer TIVA safely. This risk can lead to complications especially in patients with comorbidities or comorbidities 6-8.

Despite these challenges TIVA has been successfully used in many parts of Africa especially in low-income communities. Studies have shown that TIVA is a safe and effective anesthetic method with lower rates of postoperative nausea and vomiting compared with inhaled anesthetics. However more research is needed to better define the use of TIVA in resource-limited settings as well as strategies to address the challenges of equipment availability and the treatment and training needed by health care providers.

Access to safe and effective anesthesia is a significant challenge in many rural regions of Sub-Saharan Africa. Traditional anesthesia techniques, such as inhalational anesthesia using halothane, can be difficult to implement due to limitations in equipment, infrastructure, and resources 9-12. Total intravenous anesthesia (TIVA) has emerged as a potential solution, offering advantages such as improved hemodynamic stability and faster emergence from anesthesia.

In this report, we describe the use of TIVA in 25 cases of thyroid surgery performed in a rural region of Sub-Saharan Africa. We also discuss the benefits and potential drawbacks of TIVA anesthesia in this setting.

There is limited literature on the use of TIVA in sub-Saharan Africa and most studies have been conducted in urban hospitals. However, there is increasing interest in the potential use of TIVA in rural areas because of its simplicity and ease of administration.

Studies have shown that TIVA is a safe and effective method of maintaining anesthesia for a variety of surgical procedures and has many advantages over inhalational anesthesia. TIVA results in a faster recovery time reduces the incidence of postoperative nausea and vomiting and reduces the risk of environmental degradation compared to inhalation anesthesia. TIVA has also been shown to be an effective method of managing anesthesia in resource-limited settings with studies in Asia and Africa reporting successful use of TIVA in resource-limited settings 13-17.

But TIVA also has some drawbacks. TIVA requires the use of expensive challenging drugs in a low-resource setting. Furthermore, TIVA is difficult to titrate which increases the risk of overdose or underdosing. Additionally, TIVA requires continuous monitoring of the patients' vital signs and level of consciousness which can be difficult in resource-limited settings.

The aim of this study was to evaluate the safety and efficacy of TIVA in a rural setting in sub-Saharan Africa, focusing on a case series of 25 patients undergoing surgery with TIVA. We also aimed to discuss the potential benefits and drawbacks of TIVA in this setting, as well as the challenges of implementing this method in resource-limited areas.

## Methods

The study population consisted of 25 female patients between the ages of 24 and 69 years old who underwent thyroid surgery using TIVA. The surgical procedures performed included 10 right hemithyroidectomies, 6 subtotal thyroidectomies, 3 left hemithyroidectomies, 1 total thyroidectomy, and 3 removals of cysts.

The TIVA anesthesia protocol involved the placement of two peripheral IV catheters (20G and 18G) and induction bolus with propofol (2.5 mg/kg), bolus of fentanyl (100 mcg), rocuronium (50 mg) and morphine (5 mg) administered intravenously. Maintenance of anesthesia was achieved through a propofol infusion initiated at 2.5 mg/ kg/h and titrated according to the patient's hemodynamic response. Recovery from anesthesia was facilitated by administration of sugammadex (200 mg per patient).

Inclusion criteria for surgery included a joint visit by the surgical and anesthesia teams to determine the suitability the surgery, with consideration given to the social context to determine the size and type of surgery. An ultrasound of the neck was also performed to locate the thyroid lesion or lesions, and a thyroid hormone analysis was performed to rule out hyperthyroidism.

In this report of 25 cases of intravenous anesthesia patients underwent a 2-h recovery period after anesthesia to prevent recording and respiratory complications. The patient was admitted within 24 hours of recovery. This approach is consistent with recommended practices for postoperative care of patients undergoing thyroid surgery which may include monitoring for voice changes and dyspnea in the immediate postoperative period.

A study by Papavramidis et al. demonstrated the importance of monitoring and managing postoperative complications of thyroid surgery including airway obstruction and vocal cord paralysis. Another study by Sosa et al. It was found that a significant proportion of thyroid surgery patients experience temporary or permanent voice changes. A 2-hour recovery period and 24-hour dosing after administrationof anesthesia reduces the risk of postoperative complications and allows the patient to be properly monitored and cared for.

## Results

The duration of the hemithyroidectomies ranged from 1.5 to 2.5 hours, with an average of 2 hours. The subtotal and total thyroidectomies had a longer duration, ranging from 3 to 4.5 hours, with an average of 4 hours. Between 4 and 6 surgeries were performed per day, with the fewest surgeries performed on the first day and the most surgeries performed on the fifth day.

The 25 patients who underwent TIVA in this study were all female, with ages ranging from 24 to 69 years old. The surgical procedures performed included 10 right hemithyroidectomies, 6 subtotal thyroidectomies, 3 left hemithyroidectomies, 1 total thyroidectomy, and 3 removals of cysts.

Patients had a mean height of 165 cm +/- 5cm and a mean weight of 65 +/- 8kg kg, corresponding to a mean calculated BMI of 23.1 kg/m2. The estimated mean ideal weight for our cohort of patients would be between 58.2 and 64.3 kg, although actual weight values were used for the dose calculations given the suitability of the patients as well as the possibility of changing the doses of the farmacs

None of the patients were drug users, except for low doses of alcohol, or injecting drug users. None of the patients had active hyperthyroidism or any concomitant infection.

The ASA of the patients was between 1 and 2, with 68% of the patients being ASA I and 32% ASA 2. Those patients above or equal to 3 were discarded due to the non-ideality of the anaesthetic environment for the management of these patients. Although it is true that the correct determination of the ASA in these patients was a challenge, given that in most cases w did not have access to recent or old blood tests, as well as the impossibility of having tumour extension studies in the cases of suspected thyroid neoplasms.

Over the course of the surgical campaign, the number of surgeries varied from day to day. The first day had the fewest number of surgeries, with only 3 patients operated on. The day with the highest of surgeries was the fifth day, with 8 surgeries and 7 patients operated on.

There was only one surgical complication, which involved arterial bleeding and required re-operation. Overall, the use of TIVA anesthesia in this study demonstrated promising results for use in rural regions of Sub-Saharan Africa, with careful consideration of the specific patient population and surgical procedures.

**Figure:**
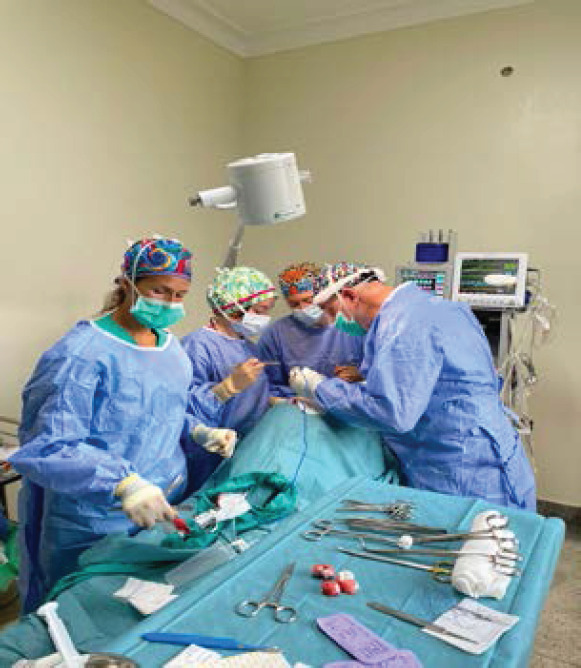
Surgery room in Holy Innocents Hospital, Kamutur, Uganda

**Figure:**
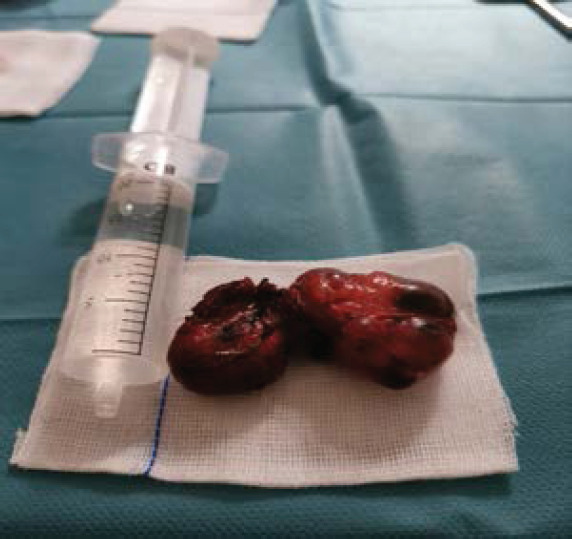
Extracted Thyroid. 420gr

## Discussion

The use of TIVA in this study shows promise for use in rural sub-Saharan Africa where conventional inhalation anesthesia is difficult to perform due to equipment infrastructure and resource constraints. Benefits of TIVA anesthesia includes improved hemodynamic stability faster emergence from anesthesia and reduced risk of environmental contamination.

However, TIVA also has potential drawbacks such as higher cost compared to conventional anesthesia methods. The specific patient population and surgical procedure should be carefully considered to determine whether the benefits of TIVA anesthesia outweigh the potential drawbacks.

The use of total intravenous anesthesia (TIVA) has become a common practice in many parts of the world, including in rural regions of sub-Saharan Africa 1. While the advantages of TIVA, such as quick induction, rapid emergence, and the ability to titrate the dose to the individual patient, make it an attractive option in low-resource settings, there are also potential challenges associated with its use.

One of the main challenges with TIVA in low-resource settings is the availability and reliability of equipment, including infusion pumps and drug monitoring devices. Without these devices, it can be difficult to ensure accurate dosing and minimize the risk of drug-related complications. In addition, the lack of trained personnel can also be a challenge, as TIVA requires skilled anesthesiologists who are knowledgeable in pharmacokinetics and drug interactions.

Anesthesia using inhalers in resource-limited settings can present significant challenges due to unreliable power supply erratic oxygen supply and lack of maintenance of anesthetic machines. Additionally the use of older generation anesthesia increases the risk of adverse events such as hypoxic respiratory depression and unstable drug overdose especially in rural areas. During our investigation we encountered problems such as severe gas leakage in the ventilator system insufficient oxygen supply and intermittent power outages. These limitations highlight the need for more reliable and safer anesthesia delivery systems in resource-poor settings.

Another potential issue with TIVA is the need for post-anesthesia care units (PACUs) to monitor patients for a longer period of time after surgery compared to surgery using other anesthetic techniques. During the process of creating the demonstrated protocol, other more multimodal approaches were considered, such as the implementation of deep cervical blocks with low doses of ketamine and fentanyl, or even the placement of cervical epidural anesthesia. While it is true that these anesthetic models would potentially have a faster and safer recovery, the lack of means as well as the technical difficulty for implementation and maintenance, rule out these anesthetic options.

In the case of the present study, patients were kept in the PACU for 2 hours post-operatively, to prevent the occurrence of post-operative respiratory and phonation problems, before being admitted to the hospital for 24 hours. However, in low-resource settings, the availability of PACUs may be limited, making it more difficult to provide adequate post-operative care. 7-9

The use of sugammadex was included in our protocol for several reasons. In the first place, our only option as a neuromuscular relaxant was rocuronium, which has a highly variable and hardly predictable half-life, we had a lack of any type of neuromuscular monitoring, and, furthermore, we had a very ample supply of sugammadex, not only in relation to the budgeted needs by the anesthesia team, but by hand from existing remnants of old expeditions. With all this, the use of sugammadex, despite the existence of neostigmine, was taken as preferable due to the safety guarantees it offered in this specific case.

Despite these challenges this study demonstrates that TIVA can be successfully applied to a variety of surgical procedures in rural sub-Saharan Africa. However close attention must be paid to equipment and personnel requirements and postoperative care to ensure optimal results.

In general, the use of TIVA in low-resource settings requires a balance between benefits and challenges and its success. This is highly dependent on the availability of adequate resources and trained personnel.

## Conclusion

TIVA offers a potential solution for providing safe and effective anesthesia in rural regions of Sub-Saharan Africa. Further research in this area may be beneficial in order to more fully understand the potential benefits and drawbacks of TIVA in these settings.
